# Supplementary material for: Diet and Respiratory Infections: Specific or Generalized Associations?
Source: Nutrients. 2022 Mar 11;14(6):1195. doi: 10.3390/nu14061195 (PMC8954090; doi:10.3390/nu14061195)
Supplement: Supplementary file 1 [file nutrients-14-01195-s001.zip › nutrients-1541331-supplementary.pdf]

Supplemental Methods

COVID-19 Diagnosis, Sample, and Statistical Analysis (Vu et al [1])

COVID-19 (SARS-CoV-2) test results from Public Health England have been dynamically linked to the UKB beginning March 16, 2020. The regularly updated COVID-19 data table provided to UKB researchers included participant ID, record date, test location (mouth, nose, throat, trachea etc.), testing laboratory (71 labs listed) and test results (negative or positive). Because no COVID-19 test data were available for UKB assessment centers in Scotland and Wales, only participants in England were included. For this analysis, we included participants with test results between March 16 and November 30, 2020, which was before vaccines were rolled out in the UK (December 8, 2020). The final study sample included 37,988 UKB participants. Our outcome of interest was whether a person had any confirmed COVID-19 infection (defined as having any positive PCR test result for SARS-CoV-2). Our exposures of interest included breastfed as a baby and consumption of coffee, tea, oily fish, processed meat, red meat, fruit, and vegetables and were defined and examined for associations with COVID-19 in the same manner described for pneumonia/influenza (see main Methods). A third model, however, further adjusted for COVID-19 exposure score (not available for pneumonia/influenza). Results from Model 2 were presented in Table 2 for the comparison to diet-pneumonia associations.

Supplementary Table S1. Candidate SNP Selection[a]

Chr:pos (b38)	Closest gene(s)	SNP[b], EA/OA	EAF				Trait	Effect
			AFR	AMR	ASN	EU		
**COVID-19 [2-7]**								
3:45796521	*SLC6A20*	rs2271616, T/G	0.03	0.12	0.07	0.13	Susceptibility	+
3:45823240	*LZTFL1*	rs10490770, C/T	0.0	0.05	0.0	0.09	Severity	+
3:101705614	*RPL24*	rs11919389, T/C	0.74	0.73	0.69	0.68	Susceptibility	+
9:133274084	*ABO*	rs529565, C/T	0.38	0.23	0.42	0.37	Susceptibility	+
9:133255928		rs8176747, C/G	0.85	0.94	0.80	0.92	ABO haplotype &	+
9:133255929		rs41302905, C/T	1.00	0.98	1.00	0.97	Blood type[c]	
9:133255935		rs8176746, T/G	0.15	0.06	0.20	0.08		
9:133257521		rs8176719, T/TC	0.73	0.76	0.58	0.61		
12:112919388	*OAS1*	rs10774671, A/G	0.41	0.70	0.78	0.65	Severity	+
19:4719431	*DPP9*	rs2109069, A/G	0.20	0.27	0.15	0.31	Severity	+
19:48867352	*PLEKHA4*	rs4801778, G/T	0.83	0.88	0.98	0.81	Susceptibility	+
21:33242905	*IFNAR2*	rs13050728,T/C	0.26	0.45	0.62	0.36	Severity	+
**Pneumonia[8-10]**								
3:151584294	*SUCNR1*	rs11708673, T/A	0.85	0.85	0.93	0.83	Susceptibility	+
6:31451033	*HLA*	rs3131623, T/A	0.04	0.09	0.02	0.11	Susceptibility	+
**Caffeine, coffee, tea [11]**								
7:17244953	*AHR*	rs4410790, C/T	0.47	0.42	0.37	0.62	Coffee, tea intake	+
							Plasma caffeine	-
15:74735539	*CYP1A2*	rs2472297, T/	0.02	0.09	0.0	0.27	Coffee, tea intake	+
							Plasma caffeine	-

Abbreviations: EA, effect allele; OA, other allele; EAF, effect allele frequency
[a]COVID-19 severity SNP rs1819040 (KANSL1) was not available in UKB, nor were any valid proxies.
[b]All SNP quality info scores >0.98.
[c]ABO haplotypes initially derived using rs8176719 (O), rs8176747 (A) and rs8176746 (B)[12]. rs41302905 was then used to verify 'BO' haplotype among those not assigned a haplotype using first 3 SNPs[5]. 10 participants (likely AO or BO) were not assigned a haplotype. Haplotypes were then used to assign blood type (phenotype): A, B, AB or O.

Supplementary Table S2. Dietary Behaviors and Risk of Pneumonia (n=470,853)

Dietary Behavior	Model 1		Model 2	
	HR (95%CI)	P	HR (95%CI)	P
**Coffee, cups/day**				
None or <1 cup	Reference		Reference	
1 cup	0.91 (0.87, 0.95)	<.0001	0.92 (0.88, 0.96)	<.0001
2-3 cups	0.94 (0.90, 0.97)	0.001	0.94 (0.90, 0.98)	0.002
≥ 4 cups	1.04 (0.99, 1.08)	0.097	1.01 (0.96, 1.05)	0.816
**Tea, cups/day**				
None or <1 cup	Reference		Reference	
1 cup	0.87 (0.82, 0.93)	<.0001	0.89 (0.83, 0.95)	<.0001
2-3 cups	0.86 (0.82, 0.90)	<.0001	0.88 (0.84, 0.920	<.0001
≥ 4 cups	0.90 (0.87, 0.94)	<.0001	0.92 (0.88, 0.96)	<.0001
**Oily fish, servings/day**				
Q1 (0-<0.07)	Reference		Reference	
Q2 (0.07- <0.14)	0.88 (0.84, 0.93)	<.0001	0.89 (0.85, 0.94)	<.0001
Q3 and 4 (≥0.14)	0.87 (0.84, 0.92)	<.0001	0.90 (0.86, 0.94)	<.0001
**Processed meat, servings/day**				
Q1 (0-<0.07)	Reference		Reference	
Q2 (0.07- <0.14)	0.97 (0.91, 1.03)	0.294	0.97 (0.91, 1.03)	0.361
Q3 (0.14- <0.43)	1.03 (0.97, 1.09)	0.360	1.02 (0.96, 1.09)	0.550
Q4 (≥0.43)	1.08 (1.01, 1.14)	0.015	1.05 (0.99, 1.12)	0.115
**Red meat, servings/day**				
Q1 (0- <0.21)	Reference		Reference	
Q2 (0.21- <0.28)	1.00 (0.95, 1.04)	0.851	1.01 (0.96, 1.06)	0.660
Q3 (0.28- <0.35)	1.04 (0.99, 1.10)	0.087	1.05 (1.00, 1.11)	0.058
Q4 (≥0.35)	1.08 (1.03, 1.13)	0.001	1.08 (1.03, 1.13)	0.002
**Fruit (fresh/dried), servings/day**				
Q1 (0- <1.00)	Reference		Reference	
Q2 (1.00- <2.25)	0.89 (0.85, 0.94)	<.0001	0.91 (0.86, 0.95)	<.0001
Q3 (2.25- <4.00)	0.83 (0.78, 0.88)	<.0001	0.85 (0.80, 0.90)	<.0001
Q4 (≥4.00)	0.82 (0.78, 0.87)	<.0001	0.85 (0.80, 0.89)	<.0001
**Vegetables (cooked/raw), servings/day**				
Q1 (0- <0.50)	Reference		Reference	
Q2 (0.50- <0.67)	0.97 (0.93, 1.01)	0.122	1.00 (0.96, 1.04)	0.934
Q3 (0.67- <1.00)	0.96 (0.91, 1.01)	0.124	1.01 (0.96, 1.06)	0.837
Q4 (≥1.00)	0.98 (0.94, 1.03)	0.419	1.04 (0.99, 1.09)	0.095
**Breastfed as a baby**				
No	Reference		Reference	
Yes	0.97 (0.93, 1.01)	0.168	0.98 (0.94, 1.02)	0.314
Don't know	1.01 (0.96, 1.05)	0.723	1.01 (0.96, 1.05)	0.711

Abbreviation: HR, hazard ratio CI; confidence interval; Q, quartile.

[a]***Model 1****: Adjusted for Townsend deprivation index, baseline age, race, education, income, employment status, type of accommodation lived in, number of co-habitants, BMI level, smoking status, physical activity, self-rated health, cholesterol-lowering medication use, antihypertension medication use, history of diabetes, history of cardiovascular disease, and history of pneumonia. Individual diet factors assessed in separate models.*
Model 2*: Adjusted for all covariates listed in* ***Model 1****, with all diet factors included in the model (i.e. mutual adjustment).*

Supplementary Table S3. Dietary Behaviors and Risk of Influenza (n=470,853)

Dietary Behavior	OR (95%CI)	P
**Coffee, cups/day**		
None or <1 cup	Reference	
1 cup	0.99 (0.84, 1.17)	0.923
2-3 cups	0.88 (0.75, 1.03)	0.098
≥ 4 cups	1.06 (0.90, 1.25)	0.494
**Tea, cups/day**		
None or <1 cup	Reference	
1 cup	0.93 (0.72, 1.18)	0.538
2-3 cups	0.83 (0.69, 0.99)	0.035
≥ 4 cups	0.92 (0.78, 1.07)	0.272
**Oily fish, servings/day**		
Q1 (0-<0.07)	Reference	
Q2 (0.07- <0.14)	0.82 (0.67, 0.99)	0.041
Quartiles 3 and 4 (≥0.14)	0.86 (0.71, 1.03)	0.092
**Processed meat, servings/day**		
Q1 (0-<0.07)	Reference	
Q2 (0.07- <0.14)	1.04 (0.82, 1.31)	0.768
Q3 (0.14- <0.43)	1.14 (0.90, 1.45)	0.264
Q4 (≥0.43)	1.04 (0.82, 1.32)	0.726
**Red meat, servings/day**		
Q1 (0- <0.21)	Reference	
Q2 (0.21- <0.28)	0.96 (0.80, 1.14)	0.621
Q3 (0.28- <0.35)	0.94 (0.77, 1.15)	0.541
Q4 (≥0.35)	0.93 (0.78, 1.11)	0.402
**Fruit (fresh/dried), servings/day**		
Q1 (0- <1.00)	Reference	
Q2 (1.00- <2.25)	0.95 (0.76, 1.17)	0.603
Q3 (2.25- <4.00)	0.88 (0.69, 1.12)	0.301
Q4 (≥4.00)	0.99 (0.79, 1.24)	0.954
**Vegetables (cooked/raw), servings/day**		
Q1 (0- <0.50)	Reference	
Q2 (0.50- <0.67)	0.96 (0.81, 1.14)	0.637
Q3 (0.67- <1.00)	1.02 (0.83, 1.25)	0.876
Q4 (≥1.00)	1.04 (0.88, 1.24)	0.624
**Breastfed as a baby**		
No	Reference	
Yes	0.93 (0.79, 1.08)	0.343
Don't know	0.91 (0.76, 1.09)	0.303

Abbreviation: OR, odds ratio; CI, confidence interval; Q, quartile.

[a]Adjusted for Townsend deprivation index, baseline age, race, education, income, employment status, type of accommodation lived in, number of co-habitants, BMI level, smoking status, physical activity, self-rated health, cholesterol-lowering medication use, antihypertension medication use, history of diabetes, history of cardiovascular disease, and history of pneumonia. Individual diet factors assessed in separate models.

Supplementary Table S4. Baseline Characteristics of the Current Analysis and the COVID-19 Analysis Samples

Baseline Characteristics[a]	Current Analysis	(Vu et al [1])
	Pneumonia analysis sample	COVID-19 analysis sample
**Number of persons**	**470,853**	**37,988**
Age, yr, mean(sd)	56.52 (8.08)	57.36 (8.23)
Female	257,048 (54.59)	20,026 (52.72)
Townsend-deprivation index, mean(sd)	-1.38 (3.04)	-1.21 (3.12)
White/British	446,903 (94.91)	35,793 (94.22)
Household Income, £< 18,000	89,786 (19.07)	8,264 (21.75)
College or university degree	155,121 (32.94)	11,000 (28.96)
Currently employed	272,358 (57.84)	20,354 (53.58)
Lived in a house	424,039 (90.06)	34,295 (90.28)
Number of co-habitants ≥ 4	89,023 (18.91)	6,821 (17.96)
Current smoker	49,677 (10.55)	4,205 (11.07)
BMI (kg/m^2), mean(sd)	27.40 (4.78)	27.93 (4.95)
Physical activity, minutes/day, mean(sd)	75.33 (96.00)	75.50 (97.52)
Poor overall health rating	20,296 (4.31)	2,412 (6.35)
Cholesterol medication use	80,719 (17.14)	8,195 (21.57)
Blood pressure medication use	96,521 (20.50)	9,610 (25.30)
History of Diabetes	24,130 (5.12)	2,680 (7.05)
History of Heart disease	26,563 (5.64)	3,003 (7.91)
Breastfed as baby	262,222 (55.69)	21,051 (55.41)
Coffee, cups/day, mean(sd)	3.27 (1.55)	2.02 (2.06)
Tea, cups/day, mean(sd)	4.12 (1.70)	3.43 (2.73)
Oily fish, servings/day, mean(sd)	0.16 (0.15)	0.16 (0.15)
Processed meat, servings/day, mean(sd)	0.21 (0.20)	0.22 (0.20)
Red meat, servings/day, mean(sd)	0.30 (0.21)	0.30 (0.21)
Fruit (fresh/dried), servings/day, mean(sd)	3.05 (2.59)	3.04 (2.60)
Vegetables (cooked/raw), servings/day, mean(sd)	0.81 (0.56)	0.82 (0.55)
Any diagnosis with pneumonia[b]	18,738 (3.76)	-
Any diagnosis with influenza[b]	1,120 (0.24)	-
COVID-19 infection[c]	-	6,482 (17.06)

[a]Values are numbers (%) unless stated otherwise.
[b]Any diagnosis in the hospital database or death records from baseline to Dec 31, 2019.
[c]Any confirmed COVID-19 infection (defined as having any positive PCR test result) between March 16 and November 30, 2020.

Supplementary Table S5: Dietary Behaviors and Risk of Pneumonia, Stratified by Sex

Dietary Behavior	Male (n=213,805)		Female (n=257,048)	
	OR (95%CI)	P	OR (95%CI)	P
**Coffee, cups/day**				
None or <1 cup	Reference		Reference	
1 cup	0.89 (0.84, 0.94)	<.0001	0.95 (0.89, 1.01)	0.096
2-3 cups	0.95 (0.90, 1.01)	0.079	0.92 (0.86, 0.98)	0.006
≥ 4 cups	1.04 (0.98, 1.10)	0.246	0.96 (0.90, 1.03)	0.301
**Tea, cups/day**				
None or <1 cup	Reference		Reference	
1 cup	0.87 (0.80, 0.95)	0.002	0.91 (0.82, 1.00)	0.056
2-3 cups	0.89 (0.83, 0.95)	<.0001	0.87 (0.81, 0.93)	<.0001
≥ 4 cups	0.91 (0.86, 0.97)	0.004	0.92 (0.86, 0.98)	0.011
**Oily fish, servings/day**				
Q1 (0-<0.07)	Reference		Reference	
Q2 (0.07- <0.14)	0.89 (0.83, 0.96)	0.001	0.88 (0.82, 0.96)	0.002
Q3 and 4 (≥0.14)	0.89 (0.84, 0.96)	0.001	0.91 (0.85, 0.98)	0.017
**Processed meat, servings/day**				
Q1 (0-<0.07)	Reference		Reference	
Q2 (0.07- <0.14)	0.93 (0.84, 1.04)	0.197	0.99 (0.91, 1.07)	0.790
Q3 (0.14- <0.43)	1.01 (0.91, 1.13)	0.810	1.00 (0.92, 1.09)	0.978
Q4 (≥0.43)	1.04 (0.94, 1.16)	0.467	1.04 (0.95, 1.14)	0.359
**Red meat, servings/day**				
Q1 (0- <0.21)	Reference		Reference	
Q2 (0.21- <0.28)	1.01 (0.94, 1.09)	0.781	1.00 (0.93, 1.07)	0.989
Q3 (0.28- <0.35)	1.04 (0.96, 1.12)	0.329	1.09 (1.01, 1.18)	0.033
Q4 (≥0.35)	1.09 (1.02, 1.17)	0.012	1.08 (1.01, 1.16)	0.025
**Fruit (fresh/dried), servings/day**				
Q1 (0- <1.00)	Reference		Reference	
Q2 (1.00- <2.25)	0.94 (0.88, 1.00)	0.057	0.85 (0.77, 0.93)	<.0001
Q3 (2.25- <4.00)	0.90 (0.83, 0.97)	0.005	0.79 (0.72, 0.87)	<.0001
Q4 (≥4.00)	0.91 (0.85, 0.98)	0.016	0.78 (0.71, 0.85)	<.0001
**Vegetables (cooked/raw), servings/day**				
Q1 (0- <0.50)	Reference		Reference	
Q2 (0.50- <0.67)	0.98 (0.93, 1.04)	0.463	1.03 (0.96, 1.10)	0.457
Q3 (0.67- <1.00)	1.00 (0.93, 1.07)	0.926	1.00 (0.92, 1.08)	0.928
Q4 (≥1.00)	1.01 (0.95, 1.07)	0.732	1.06 (0.98, 1.14)	0.149
**Breastfed as a baby**				
No	Reference		Reference	
Yes	0.96 (0.91, 1.02)	0.221	0.99 (0.93, 1.04)	0.608
Don't know	1.00 (0.94, 1.07)	0.926	1.00 (0.93, 1.07)	0.949

Abbreviation: OR, odds ratio; CI, confidence interval; Q, quartile.

[a]***Model 1****: Adjusted for Townsend deprivation index, baseline age, race, education, income, employment status, type of accommodation lived in, number of co-habitants, BMI level, smoking status, physical activity, self-rated health, cholesterol-lowering medication use, antihypertension medication use, history of diabetes, history of cardiovascular disease, and history of pneumonia. Individual diet factors assessed in separate models.*
Model 2*: Adjusted for all covariates listed in* ***Model 1****, with all diet factors included in the model (i.e. mutual adjustment).*

Supplementary Table S6. Candidate Genetic Factors and Risk of Pneumonia/COVID-19 Infection

**Pneumonia (n=335,205)**		
SNP_EA	OR (95% CI)	P
rs11708673_T	1.05 (1.01, 1.08)	0.008
rs3131623_T	0.98 (0.95, 1.01)	0.151
Blood type		
O	Reference	
A	0.98 (0.94, 1.02)	0.228
AB	1.00 (0.91, 1.11)	0.980
B	1.03 (0.96, 1.10)	0.383
**COVID-19 infection (n=26,919)**		
SNP_EA	OR (95% CI)	P
rs2271616_T	1.20 (1.12, 1.28)	<.0001
rs10490770_C	1.09 (1.00, 1.20)	0.056
rs11919389_T	1.10 (1.04, 1.15)	0.000
rs529565_C	1.09 (1.03, 1.14)	0.001
rs10774671_A	1.04 (0.99, 1.09)	0.175
rs2109069_A	1.04 (0.99, 1.09)	0.137
rs4801778_G	1.00 (0.94, 1.06)	0.973
rs13050728_T	1.06 (1.00, 1.11)	0.035
Blood type		
O	Reference	
A	1.11 (1.03, 1.19)	0.004
AB	1.22 (1.01, 1.46)	0.038
B	1.04 (0.91, 1.17)	0.595

Abbreviation: OR, odds ratio; CI, confidence interval; EA, effect allele; SNP, single-nucleotide-polymorphism

[a]*Adjusted for Townsend deprivation index, baseline age, race, education, income, employment status, type of accommodation lived in, number of co-habitants, BMI level, smoking status, physical activity, self-rated health, cholesterol-lowering medication use, antihypertension medication use, history of diabetes, history of cardiovascular disease, history of pneumonia, and all diet factors.*

Supplementary Figure S1. Flow chart depicting the study design and the analysis sample

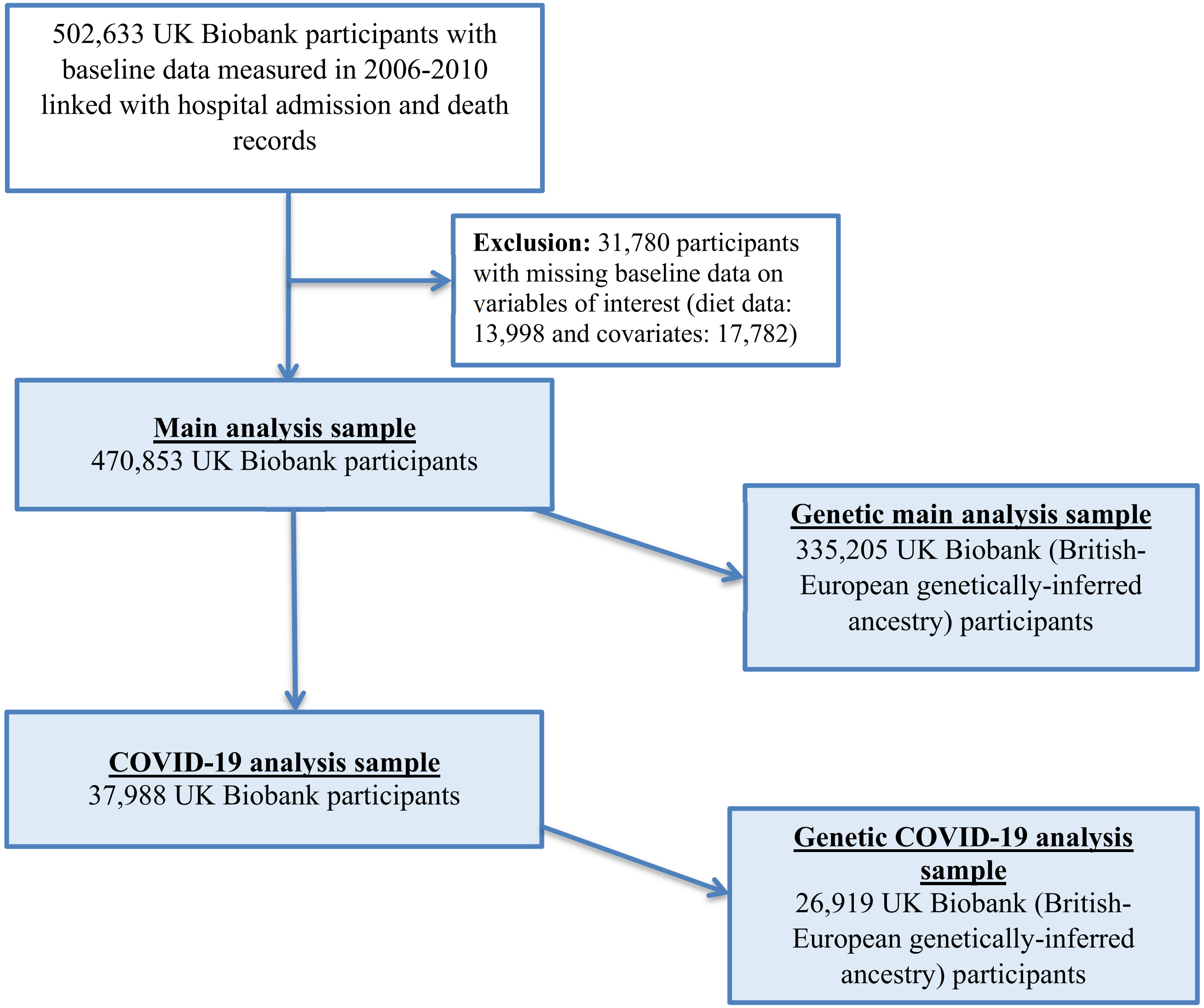

References

1. Vu, T.-H.T.; Rydland, K.J.; Achenbach, C.J.; Van Horn, L.; Cornelis, M.C. Dietary Behaviors and Incident COVID-19 in the UK Biobank. *Nutrients* **2021**, *13*, 2114.
2. Covid- Host Genetics Initiative. Mapping the human genetic architecture of COVID-19. *Nature* **2021**, 10.1038/s41586-021-03767-x, doi:10.1038/s41586-021-03767-x.
3. Pairo-Castineira, E.; Clohisey, S.; Klaric, L.; Bretherick, A.D.; Rawlik, K.; Pasko, D.; Walker, S.; Parkinson, N.; Fourman, M.H.; Russell, C.D., et al. Genetic mechanisms of critical illness in COVID-19. *Nature* **2021**, *591*, 92-98, doi:10.1038/s41586-020-03065-y.
4. Genomewide Association Study of Severe Covid-19 with Respiratory Failure. *New England Journal of Medicine* **2020**, *383*, 1522-1534, doi:10.1056/NEJMoa2020283.
5. Shelton, J.F.; Shastri, A.J.; Ye, C.; Weldon, C.H.; Filshtein-Sonmez, T.; Coker, D.; Symons, A.; Esparza-Gordillo, J.; Chubb, A.; Fitch, A., et al. Trans-ancestry analysis reveals genetic and nongenetic associations with COVID-19 susceptibility and severity. *Nature Genetics* **2021**, *53*, 801-808, doi:10.1038/s41588-021-00854-7.
6. Ellinghaus, D.; Degenhardt, F.; Bujanda, L.; Buti, M.; Albillos, A.; Invernizzi, P.; Fernández, J.; Prati, D.; Baselli, G.; Asselta, R. Severe Covid-19 GWAS Group. Genomewide association study of severe Covid-19 with respiratory failure. *N Engl j Med* **2020**, *383*, 1522-1534.
7. Bugert, P.; Rink, G.; Kemp, K.; Klüter, H. Blood group ABO genotyping in paternity testing. *Transfusion Medicine and Hemotherapy* **2012**, *39*, 182-186.
8. Campos, A.I.; Kho, P.F.; Vazquez-Prada, K.X.; García-Marín, L.M.; Martin, N.G.; Cuéllar-Partida, G.; Rentería, M.E. Genetic susceptibility to pneumonia: A GWAS meta-analysis between UK Biobank and FinnGen. *medRxiv* **2020**, 10.1101/2020.06.22.20103556, 2020.2006.2022.20103556, doi:10.1101/2020.06.22.20103556.
9. Chen, H.-H.; Shaw, D.M.; Petty, L.E.; Graff, M.; Bohlender, R.J.; Polikowsky, H.G.; Zhong, X.; Kim, D.; Buchanan, V.L.; Preuss, M.H., et al. Host genetic effects in pneumonia. *The American Journal of Human Genetics* **2021**, *108*, 194-201, doi:https://doi.org/10.1016/j.ajhg.2020.12.010.
10. Tian, C.; Hromatka, B.S.; Kiefer, A.K.; Eriksson, N.; Noble, S.M.; Tung, J.Y.; Hinds, D.A. Genome-wide association and HLA region fine-mapping studies identify susceptibility loci for multiple common infections. *Nature communications* **2017**, *8*, 1-13.
11. Cornelis, M.C.; Kacprowski, T.; Menni, C.; Gustafsson, S.; Pivin, E.; Adamski, J.; Artati, A.; Eap, C.B.; Ehret, G.; Friedrich, N., et al. Genome-wide association study of caffeine metabolites provides new insights to caffeine metabolism and dietary caffeine-consumption behavior. *Human molecular genetics* **2016**, *25*, 5472-5482, doi:10.1093/hmg/ddw334.
12. McLachlan, S.; Giambartolomei, C.; White, J.; Charoen, P.; Wong, A.; Finan, C.; Engmann, J.; Shah, T.; Hersch, M.; Podmore, C., et al. Replication and Characterization of Association between ABO SNPs and Red Blood Cell Traits by Meta-Analysis in Europeans. *PLoS One* **2016**, *11*, e0156914, doi:10.1371/journal.pone.0156914.
